# Production and Physicochemical Characterization of the Gel Obtained in the Fermentation Process of Blue Corn Flour (*Zea mays* L.) with *Colletotrichum gloeosporioides*

**DOI:** 10.3390/gels10050314

**Published:** 2024-05-03

**Authors:** Guadalupe Villarreal-Rodríguez, Jesús Manuel Escajeda-García, Lingyun Chen, Nubia Amaya-Olivas, Teresita Ruiz-Anchondo, David Neder-Suarez, David Chávez-Flores, Néstor Gutierrez-Mendez, León Hernández-Ochoa

**Affiliations:** 1 Facultad de Ciencias Químicas, Universidad Autónoma de Chihuahua (UACH), Circuito Universitario s/n, Campus II, Chihuahua 31125, Mexico; a274137@uach.mx (G.V.-R.); p274235@uach.mx (J.M.E.-G.); namaya@uach.mx (N.A.-O.); dneder@uach.mx (D.N.-S.); dchavezf@uach.mx (D.C.-F.); ngutierrez@uach.mx (N.G.-M.); 2 Department of Agricultural Food & Nutritional Science, University of Alberta, Edmonton, AB T6G 2P5, Canada; lingyun1@ualberta.ca; 3 Facultad de Ciencias Agrotecnológicas, Universidad Autónoma de Chihuahua, Av. Pascual Orozco s/n, Campus I, Santo Niño, Chihuahua 31350, Mexico; truiz@uach.mx

**Keywords:** gel, organic solvents, rheological properties, *Colletotrichum gloeosporioides*, carbohydrates, pectin

## Abstract

Food gels are viscoelastic substances used in various gelled products manufactured around the world. Polysaccharides are the most common food gelling agents. The aim of this work was the production and characterization of a gel produced in a blue corn flour fermentation process, where different proportions were used of blue corn (*Zea mays* L.) flour and Czapek Dox culture medium (90 mL of culture medium with 10 g of blue corn flour, 80 mL of culture medium with 20 g of blue corn flour, and 70 mL of culture medium with 30 g of blue corn flour) and were fermented for three different durations (20, 25, and 30 days) with the *Colletotrichum gloeosporioides* fungus. A characterization of the gel was carried out studying the rheological properties, proximal analysis, toxicological analysis, microscopic structure, and molecular characterization, in addition to a solubility test with three different organic solvents (ethanol, hexane, and ethyl acetate, in addition to water). The results obtained showed in the rheological analysis that the gel could have resistance to high temperatures and a reversible behavior. The gel is soluble in polar solvents (ethanol and water). The main chemical components of the gel are carbohydrates, especially polysaccharides, and it was confirmed by FT-IR spectroscopy that the gel may be composed of pectin.

## 1. Introduction

The definition of gels can be complex, since they are combined with elastic characteristics, typical of solids, and have the ability to flow, like a liquid [1]. Gels are a common form of soft matter that arises from the association of various small molecules, and polymeric and particulate components [2].

Gel’s formation processes can be very varied, but one of the most important is through a fermentation process, which is one of the most used techniques since ancient times to preserve food and is defined as a technique of biological conversion of complex substrates into simple compounds by various microorganisms such as bacteria and fungi. Fermentation can be carried out with different substrates, including cereals such as corn (*Zea mays* L.), which is a grass plant with its origins in the American continent and is considered one of the most important cereals worldwide, due to its exceptional geographical adaptability, expanding in Europe, Africa, and the Middle East. In Mexico, the center of origin, with the domestication and diversification of corn (*Zea mays* L.), there are 59 races according to the classification based on morphological and isoenzymatic characteristics, and from 220 to 300 races of corn exist in the American continent, among which are the pigmented variants that can be a range of colors including red, pink, blue, purple, and black [3].

Corn is a good metabolizable energy source and, despite the low protein content, it is an excellent source of carbohydrates and is considered a vital food grain [3,4]. Microorganisms are related as producers of important gelatinous substances, via fermentation, which, due to the metabolization of different macromolecules, results in substances such as carbohydrates present in foods, improving their qualities in terms of their rheological and functional properties [5,6]. Filamentous fungi are part of that group of microorganisms [7]. Fungi are eukaryotic organisms with organized nuclei, whose nuclear membrane is well defined; they are aerobic, heterotrophs, and, in general, not motiles [8]. To understand the physicochemical characteristics of a gel, it is necessary to know the rheological properties and those that are not related to them. The rheological properties depend on the fitness of the molecular network, which likely means that the way the molecular network is structured or organized determines the rheological (flow) properties of the material [9]. Measurement can be made that shows the relationship between stress (force per unit of area) and tension (deformation due to applied force) for a gel under compression. The rheological measurement of gel characteristics has been widely classified into large and small tests: the first are used to measure stress, tension and fault properties of a gel [10], and the second ones are used to define structural characteristics [11]. Therefore, there is a need for the measurement of the visco-elastic (rheological) characteristics of the material and, on the other hand, there are non-rheological methods for the measurement of the characteristics of the gels. Fundamental tests such as microscopic, molecular, and proximal analysis characterization do not depend on the geometry of the sample and the instrument used [12].

The aim of this work was the production and characterization of a gel produced in a blue corn flour fermentation process, where different proportions were used of blue corn (*Zea mays* L.) flour and Czapek Dox culture medium (90 mL of culture medium with 10 g of blue corn flour, 80 mL of culture medium with 20 g of blue corn flour, and 70 mL of culture medium with 30 g of blue corn flour), which were fermented for three different durations (20, 25, and 30 days) with the *Colletotrichum gloeosporioides* fungus. It should be noted that no literature has been found that reports the production of a gel from a fermentation of blue corn flour and a filamentous fungus, which gives the importance of innovation to this research work.

## 2. Results and Discussion

### 2.1. Fermentation of Blue Corn (Zea mays L.) Flour and Colletotrichum gloeosporioides

**Figure 1:**
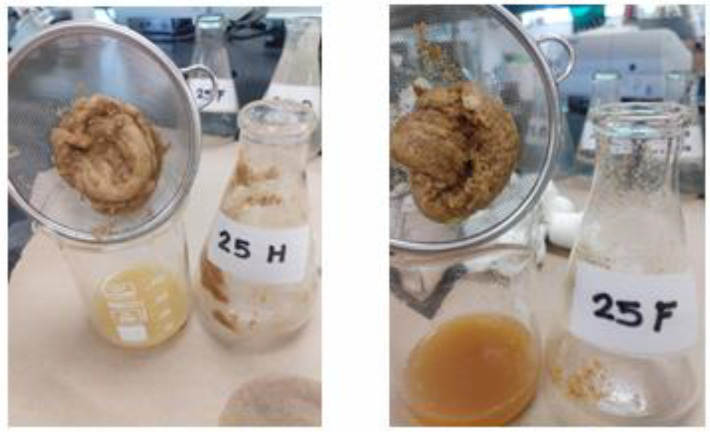
Gels obtained in the fermentation process of blue corn flour (*Zea mays* L.) with *Colletotrichum gloeosporioides*.

The concentration of the spore solutions was calculated, obtaining 1.3 × 10^6^ conidia/mL, which was the desired concentration so no dilution was required. During fermentation, two gels were obtained from samples 25F and 25H, corresponding to the proportions of 80 mL of Czapek Dox culture medium and 20 g of blue corn flour and 70 mL of Czapek Dox culture medium, respectively. Villarreal-Rodríguez et al. [12] reported the production of a single gel in the fermentation of blue corn flour and *C. gloeosporioides*, so in the present project with the same fermentation conditions it was produced again. In Figure 1, the gels obtained in the fermentation process of blue corn flour (*Zea mays* L.) with *Colletotrichum gloeosporioides* are depicted.

### 2.2. Proximal Analysis of the Gel Obtained in the Fermentation Process

The proximal analysis serves the purpose of determining the macronutrient composition of a food item, thereby revealing its physicochemical characteristics, which are influenced by various factors including climate, genetics, and soil composition [13,14]. The decision to undertake a proximal analysis of the fermentation-derived gels was driven by the imperative to ascertain the predominant chemical constituent (carbohydrates, proteins, lipids) within the samples. This pivotal step was undertaken to orient the subsequent characterization towards the identified macromolecule, thereby elucidating its primary composition within the matrix.

On analyzing the gels obtained from the fermentation of blue corn (*Zea mays* L.) flour and Czapek Dox culture medium with *Colletotrichum gloeosporioides*, it was found that carbohydrates constituted the highest percentage, accounting for 73.6 g/100 g of the chemical composition. This aligns with findings by Mex-Álvarez et al. [15], who reported a carbohydrate percentage of 74.30 g/100 g, in blue–purple corn, suggesting that carbohydrates are consistently the predominant macromolecule in such trials. The discrepancy in carbohydrate content between sample 25F and 25H stems from the higher allocation of blue corn flour in sample 25F, totaling 30 g, thus surpassing sample 25H by 10 g. Humidity content was measured at 9.19 g/100 g, while ash content was recorded at 1.79 g/100 g. In comparison, Mex-Álvarez et al. [15] reported a humidity content of 10.43 g/100 g and ash content of 1.42% in blue corn (*Zea mays* L.). Regarding lipid and protein content, the present study yielded percentages of 6.66 g/100 g, and 8.67 g/100 g, respectively, which are notably higher than the typical values found in literature reviews, where lipid content around 4 g/100 g and protein content around 6 g/100 g are commonly reported in proximal analyses of blue corn (*Zea mays* L.). The proximal analysis of the fermentation gels consistently revealed carbohydrates as the macromolecule with the highest percentage, followed by moisture content. However, there were variations in the percentage composition between the two gel samples. In the 25H gel (fermented for 25 days with 70 mL of Czapek Dox culture medium and 30 g of blue corn flour (*Zea mays* L.)), carbohydrate content was 24.43 g/100 g, while in the 25F sample (fermented for 25 days with 80 mL of Czapek Dox culture medium and 20 g of blue corn flour (*Zea mays* L.)), it was 2.56 g/100 g. The 25F sample also exhibited the highest humidity content at 84 g/100 g, in contrast to the 25H sample, which contained 63.78 g/100 g. Table 1 displays the proximal analysis of the gel samples obtained in the fermentation process of blue corn flour (*Zea mays* L.) with *Colletotrichum gloeosporioides*, along with the analysis of the flour without undergoing any fermentation process.

### 2.3. Solvent Solubility Tests

The gels derived from fermenting blue corn flour (*Zea mays* L.) with *Colletotrichum gloeosporioides* exhibited solubility in polar solvents such as distilled water (H_2_O) and ethanol (CH_3_CH_2_OH). Understanding the solubility behavior of organic compounds involves three fundamental rules. Firstly, smaller organic molecules tend to dissolve more readily in water than larger ones. Secondly, polar organic molecules, particularly those capable of hydrogen bonding, display enhanced solubility in water compared with nonpolar molecules. Thirdly, compounds in their ionic form demonstrate a greater propensity to dissolve in water than their neutral counterparts [16]. For instance, sample 2F (comprising 80 mL of Czapek Dox culture medium and 20 g of blue corn flour) yielded a pH of 6.1 ± 0.10, while sample 25H (consisting of 70 mL of Czapek Dox culture medium and 30 g of blue corn flour) recorded a pH value of 6.63 ± 0.05. pH values below 4 indicate the presence of carboxylic acid, whereas those equal to or greater than 8 suggest the presence of amines within the structure.

Furthermore, sample 25F (comprising 80 mL of Czapek Dox culture medium and 20 g of blue corn flour) exhibited bubbling when solubilized with 5% (*w*/*v*) NaHCO_3_. This bubbling resulted from a reaction between the carboxylic acids present in the gel samples and NaHCO_3_, forming water-soluble salts and releasing carbon dioxide (CO_2_). Table 2 presents the results of the solubility test conducted on the gels obtained in the fermentation process of blue corn flour with *Colletotrichum gloeosporioides*.

### 2.4. Median Lethal Dose (LD_50_) with Artemia salina

The median lethal dose of the gels produced in the fermentation of blue corn flour (*Zea mays* L.) with *Colletotrichum gloeosporioides* indicated their acute toxicity, that is, the exact concentration (mg/kg) of each gel sample necessary to be able to have a toxic effect on half of individuals after a certain time. It is important to determine if these concentrations are allowed when using them as food additives, which is the main possible application for the gels obtained from the fermentation of blue corn (*Zea mays* L.) meal with *Colletotrichum gloeosporioides*. According to the evaluation of some food additives of the Food and Agriculture Organization of the United States (FAO), there are some gelling agents for which it is very important to determine the median lethal dose of such substances to establish food safety during consumption [17]. Xanthan gum is a high molecular weight polysaccharide that is mainly made up of D-glucose and D-mannose as the dominant hexose in its structure, in addition to having D-glucuronic acid and pyruvic acid. It is produced by the fermentation of a rich carbohydrate source by the bacterium *Xanthomonas campestris*. Xanthan gum exhibited low oral toxicity, in rats, mice, and dogs, with LD_50_ values ranging from 1000–45,000 mg/kg [17]. Table 3 illustrates the determination of median lethal dose (LD50) for the gels obtained in the fermentation process of blue corn flour (*Zea mays* L.) with *Colletotrichum gloeosporioides*.

### 2.5. Analysis of Rheological Properties of the Gel Obtained in the Fermentation Process

**Figure 2:**
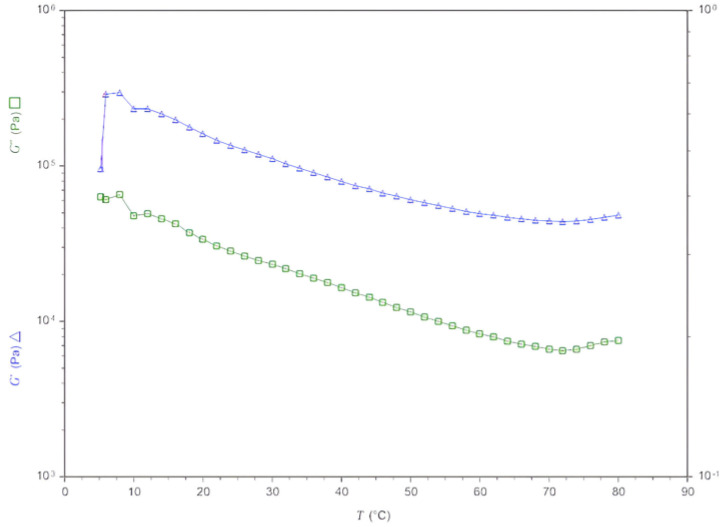
Analysis of rheological properties (G′ and G″ against temperature) of the gel obtained in the fermentation process of blue corn flour *(Zea mays* L.) and *Colletotrichum gloeosporioides*.

The gels produced in the fermentation of blue corn flour (*Zea mays* L.) with *Colletotrichum gloeosporioides* were subjected to a temperature scan, where the rheological changes produced as irreversible changes in their shape, the sensitivity they have to the temperature, and the repercussion of this in the quality and the efficiency that can contribute to a product were determined. In the graph, there are different points that describe the behavior of the gel when increasing the storage modulus (G′) and the temperature. Point #3 reached the highest G′ value, corresponding to a temperature of 7.998 °C, which indicates that under these conditions the gel retains its solid state [18]. The temperature increases to 10.005 °C and the storage modulus to 232.672 Pa, identified as point #4, then, as the temperature increases to 12.001 °C, the value of G′ increases slightly again to 232.779 Pa, at point #5. From the mentioned points, the temperature increases and the G′ decreases linearly. Between point #33 (G′: 45,001.5 Pa; T: 68.006 °C) and point #37 (G′: 45,029.2 Pa; T: 75.996 °C), a slight curve can be observed where, precisely at point #35, which is the lowest value of G′, with 44,035.2 Pa and 78.996 °C, the decrease stops and the increase of G′ begins. This demonstrates that, when the temperature increased and the value of G′ decreased, the gel was losing its solid state, becoming rubbery, but, when the value of G′ began to increase, it solidified again. At point #37, the value of G′ continues to increase as the temperature increases, which may indicate that the gel might have reversible changes, because the value of G′ continues to increase as the temperature increases, in the same way; this indicates the gel could be resistant to high temperatures. Figure 2 depicts the analysis of rheological properties, specifically the storage modulus (G′) and loss modulus (G″) against temperature, of the gel obtained through the fermentation process of blue corn flour (*Zea mays* L.) with *Colletotrichum gloeosporioides*.

### 2.6. Microscopic Characterization

The morphology of the starch granules present in the corn was observed in the microphotographs of the gels obtained in the fermentation process. Di Paola et al. [19] evaluated the gelatinization of corn starch at different temperatures for its subsequent hydrolysis with enzymatic methods. They demonstrated by means of microphotographs in an optical microscope with a 40× objective that morphological changes were observed as the temperature increased in the granules and there was a decrease in birefringence (presence of a “Maltese cross” in the center of the granules) until granular rupture from 65 °C.

**Figure 3:**
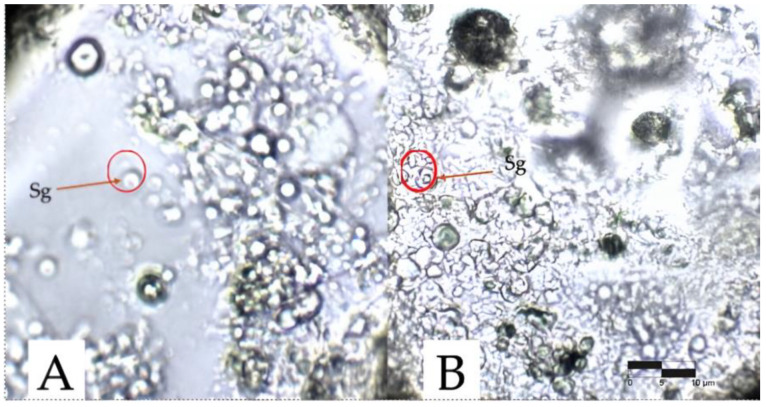
Microscopic characterization of the gels from the fermentation of blue corn flour (*Zea mays* L.) and *Colletotrichum gloeosporioides*. Sg means granules of starch. (**A**) Sample 25H (80 mL of Czapek Dox culture medium and 20 g of blue corn meal (*Zea mays* L.)). (**B**) Sample 25F (80 mL of Czapek Dox culture medium and 20 g of corn meal blue (*Zea mays* L.)).

In the microphotograph depicted in Figure 3, analysis of the two gel samples reveals an absence of the previously mentioned characteristics associated with gelatinization. Notably, granules retain their structural integrity and exhibit persistent birefringence. This significant observation negates starch gelatinization as the primary determinant for gel formation resulting from the fermentation of blue corn (*Zea mays* L.) flour with *Colletotrichum gloeosporioides*.

### 2.7. Structural Characterization with Fourier Transform Infrared Spectroscopy (FT-IR)

**Figure 4:**
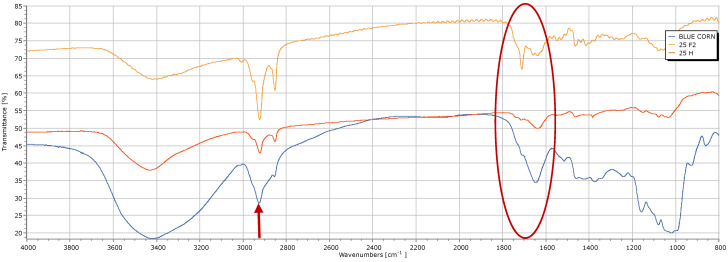
FTIR spectra of the 25F, 25H, and blue cornmeal (*Zea mays* L.) gel samples.

The prominent band observed at 3400 cm^−1^ in the spectra of the 25F gel of the Figure 4, likely arises from the stretching vibration of O-H bonds, suggesting the presence of a carboxylic acid, similar to how it would indicate N-H stretching vibrations in amine salts. Furthermore, the band at 1711 cm^−1^ is likely attributed to the C=O stretching vibration of a carboxylic acid, while the absorption band at 1639 cm^−1^ may correspond to C=C bending. Similarly, the broad band at 2922 cm^−1^ in the 25H gel spectrum suggests the presence of a carboxylic acid, consistent with the N-H stretching vibrations observed in amine salts, as noted previously in sample 25F. Additionally, the band at 2853 cm^−1^ could also be attributed to the O-H stretching vibration of a carboxylic acid, while another band at 1640 cm^−1^ may indicate C=C bending of alkenes. To facilitate comparison of functional groups present in the gel samples, blue corn flour (*Zea mays* L.) was subjected to FTIR spectroscopy. The broad band at 3440 cm^−1^ in the blue corn flour samples suggests O-H stretching vibrations typical of alcohols. Moreover, the absorption band at 1647 cm^−1^ may indicate C=C bending of alkenes, while the band at 1372 cm^−1^ could be attributed to C=O stretching vibrations of carboxylic acids. When comparing these findings with those reported by Joel et al. [20], similarities emerge, such as the band at 3438 cm^−1^ associated with O-H stretching vibrations of alcohols, and the band at 2923.9 cm^−1^ (red arrow) indicating O-H stretching vibrations of carboxylic acids. Furthermore, the band at 1750 cm^−1^ (red oval circle) corresponds to the C=O stretching vibrations of esters, while the band at 1636 cm-1 indicates C=C stretching vibrations of alkenes, and the band at 1016.2 cm^−1^ is attributed to the C-N stretching vibrations of amines. These observations confirm that the gel samples likely consist of polysaccharides, particularly pectin, as indicated by the similarity in absorption patterns to those of commercial pectin. This similarity is further supported by the presence of carboxylic acids, which are characteristic functional groups of polysaccharide structures. Additionally, the extensive presence of hydroxyl groups, as highlighted in the literature [18], contributes to the hydrophilic nature of these compounds, facilitating their ability to form viscous dispersions or gels in aqueous environments.

### 2.8. Factorial Analysis of the Fermented Samples

The value of F of the factor of the proportions, time, and interaction between both factors is less than the critical F corresponding to the value of α (significance level) of 0.05. Therefore, the null hypothesis was not rejected for either of the two factors and their interaction, resulting in the proportions, time, or interaction between them not having an influence on the amount of gel produced in the fermented samples. All data collected from the factorial design are compiled in Table 4.

## 3. Conclusions

The gel was synthesized through the fermentation process of blue corn (*Zea mays* L.) with *Colletotrichum gloeosporioides* over a span of 25 days. Two distinct samples were generated using proportions of 80 mL of Czapek Dox culture medium and 20 g of blue corn flour (*Zea mays* L.), and 70 mL of Czapek Dox culture medium and 30 g of blue corn (*Zea mays* L.) flour. Carbohydrates emerge as the predominant biomolecule in the chemical composition of both blue corn flour (*Zea mays* L.) and the gels produced during fermentation. In the LD_50_ analysis conducted with *Artemia salina*, the gels exhibited low food toxicity at minimal concentrations, in contrast to other gelling agents such as xanthan gum. Solvent solubility tests revealed that the gels produced during fermentation displayed solubility in polar solvents, indicating the presence of molecules within the gels, with bonds between atoms possessing significantly different electronegativities. Furthermore, rheological analysis demonstrated reversible changes in the gel at elevated temperatures, suggesting diverse potential applications. These applications span the computer industry, where the gel could form components of flexible mobile devices, the data storage sector, and the electronic industry for battery production and energy conversion. Additionally, the gel exhibits intriguing biocompatibility with the human body, facilitating its use in medical devices for physiological monitoring among other medical applications. Factorial design experiments affirmed that proportions, fermentation time, or their interaction significantly influence gel production during fermentation. This research underscores that the gels derived from the fermentation of blue corn flour (*Zea mays* L.) may predominantly consist of polysaccharides, especially pectin, thus offering a promising alternative source for commercial purposes and serving as a potential raw material for the food industry.

## 4. Materials and Methods

### 4.1. Complete Fermentation Process

#### Blue Corn Flour Preparation

It was imperative for the grains to undergo a thorough cleaning process followed by sterilization. The blue corn grains were carefully placed in clean and sterile jars, which were then transferred to the All American autoclave (Model ALL-1941X, Wisconsin Aluminum Foundry Co., Inc., Manitowoc, WI, USA) and subjected to a temperature of 120 °C for 15 min. Subsequently, the blue corn grains were ground and weighed, ensuring that all procedures were carried out under sterile conditions.

### 4.2. Culture Maintenance and Inoculum Preparation

The fungus *C. gloeosporioides* was originally isolated from a fruit called Chagalapoli (*Ardissia compressa*) by Alarcón-Sáenz [21]. Subsequently, it was cultured on sterilized potato dextrose agar (PDA) at 28 °C for 8 days, with subsequent transfers to new culture media as needed. To prepare the fungal spore suspension, the method described by Colomé et al. [22] was employed. This involved cultivating *C. gloeosporioides* for 10 days on Petri dishes containing PDA medium supplemented with 20% *w*/*v* potato infusion, 0.2% *w*/*v* dextrose, and 0.2% *w*/*v* agar. A sterile solution of Tween 80 (C_64_H_114_O_26_) at 1% *v*/*v* was added, followed by manual agitation for 2 min. Subsequently, 1 mL of this solution was withdrawn and placed in a Neubauer chamber (Tiefe-Depth Profondeur 0.100 mm, Superior Marienfeld, Lauda-Königshofen, Germany) for observation. The spores were then counted using a Zeiss Primo Star 2 optical microscope (Jena Göttingen, Germany) at 10× and 40× magnifications.

### 4.3. Fermentation Process

Three different proportions were devised, where the amounts of culture medium and blue corn flour were varied. These proportions were labeled as ABC samples (consisting of 90 mL of Czapek Dox culture medium and 10 g of blue corn flour), DEF samples (comprising 80 mL of Czapek Dox culture medium and 20 g of blue corn flour), and GHI samples (comprising 70 mL of Czapek Dox culture medium and 30 g of blue corn flour). Additionally, the fermentation time was adjusted to 20, 25, and 30 days for each set of samples. The fungal spore solution inoculations were conducted in a sterile laminar flow hood equipped with a UV lamp (UVP, LLC Ultra-Violet Products Ltd., Upland, CA, USA), which was pre-activated for 30 min. Subsequently, the samples were fermented in an Innova 4300 Shaker Incubator (New Brunswick Scientific Company, Inc., Edison, NJ, USA) at a temperature of 28 °C and 50 rpm for the specified durations (20, 25, and 30 days).

### 4.4. Characterization of the Gel Obtained in the Fermentation

#### 4.4.1. Solvent Solubility Tests

A total of 200 mg of each triplicate gel sample (25G and 25H) was dissolved in 5 mL of hexane (C_6_H_14_), ethyl acetate (C_4_H_8_O_2_), ethanol (C_2_H_6_O), and distilled water (H_2_O). Each sample was then vortexed for 1–2 min. The solvent where the gel samples dissolved completely was chosen. The pH of the 25H and 25F gel samples was determined. Solutions from the 10% *m*/*v* gels were prepared. The pH of the samples was measured with the Thermo-Scientific Orion 3-Star potentiometer (Thermo Fisher Scientific, Waltham, MA, USA): 25 mg of gel sample was placed in a test tube, and 500 µL of 5% *m*/*v* sodium bicarbonate was added. The assay was performed in triplicate.

#### 4.4.2. Median Lethal Dose (LD_50_) with *Artemia salina*

The hatching of *Artemia salina* eggs was carried out by depositing 0.1 g of the eggs in an Erlenmeyer flask of 1 L volume with a 38% *m*/*v* saline solution in dark conditions, with an oxygen supply and at 25 °C. After 24 h, the eggs were transferred to another flask with the same conditions of darkness, oxygenation, and temperature for another 24 h. For the preparation of the solutions with the gels, they were diluted in distilled water to obtain concentrations of 100, 75, 50, 25, and 10 mg/kg. After 24 h, 10 nauplii were placed in sterile glass vials, to later add 3 mL of the gel solutions to be tested. A 38% *m*/*v* saline solution was used as a negative control. After 24 h in dark conditions, the dead nauplii were counted with the help of a stereoscopic microscope ST-30 (Optika Italy Scientific company, Ponteranica, Italy) The LD_50_ was determined with the probit method in Minitab software v.17.1.0 (Minitab LLC, State College, PA, USA).

#### 4.4.3. Analysis of Rheological Properties

A temperature sweep was made with a Discovery HR-3 rheometer (TA Instruments, New Castle, DE, USA) of the gels produced in the fermentation process. The instrument used was a 40 mm steel parallel plate. The gel samples were subjected to a temperature scan from 25 °C to 98 °C, with a constant 1% shear stress, frequency of 1 hz, and a gap of 1000 μm. Storage modulus (G′), loss modulus (G″), and phase angle (δ) were plotted against temperature. The data were obtained using the TRIOS^®^ software version 5.4 (T. A. instruments, New Castle, DE, USA).

#### 4.4.4. Microscopic Characterization

The fresh samples of the gels were visualized at 25F (80 mL of Czapek Dox culture medium and 20 g of blue corn flour (*Zea mays* L.)) and 25H (70 mL of Czapek Dox culture medium and 30 g of blue corn flour (*Zea mays* L.)), and were was observed at 10 and 40 magnifications in an optical microscope (Optika Scientific Company, Ponteranica, Italy).

#### 4.4.5. Structural Characterization with Fourier Transform Infrared Spectroscopy (FT-IR)

Samples of the gels and blue corn (*Zea mays* L.) meal were dried in a Thermo Scientific Isotemp^®^ vacuum drying oven (Thermo Fisher Scientific, Waltham, MA, USA) at a pressure of 1 mmHg for 48 h. The gel samples were lyophilized in a E-9320 freeze dyer (LABCONCO, Kansas City, MO, USA) at a temperature of −45 °C and a pressure of 170 × 103 Bar for 48 h. Pellets were made with a specialized hydraulic manual press, and consisted of potassium bromide (KBr, CAS 7758-02-3, grade FT-IR Sigma Aldrich, St. Louis, MO, USA), previously dried at 50 °C for 48 h, and the gel samples, for subsequent analysis in FT-IR. The KBr-sample gel pellets were deposited directly on the equipment. Infrared transmission spectra of the pellets were recorded at room temperature using a Thermo-Scientific Nicolet 6700 spectrometer (Thermo Fisher Scientific, Waltham, MA, USA) at 2 cm^−1^ resolutions in the 800–4000 cm^−1^ range, using methodology modified from Sudhamani et al. [23].

#### 4.4.6. Statistical Analysis

The statistical analysis involved a factorial design incorporating two main factors: fermentation time (in days) and the proportions of ingredients used (specifically, the quantity of blue corn flour in grams and the volume of Czapek Dox culture medium in milliliters), as well as the interaction between these factors. The response variable under consideration was the quantity of gel produced during the fermentation process of blue corn flour (*Zea mays* L.) with *Colletotrichum gloeosporioides*. For each factor combination, various statistical parameters were computed, including the sum of squares (SS), degrees of freedom (DF), mean squares (MS), F-value, and the critical F-value corresponding to a significance level (α) of 0.05. All analyses were performed using the statistical software Minitab v.17.1.0 (Minitab, LLC, State College, PA, USA).

## Figures and Tables

**Table 1:** Proximal analysis of the gel samples obtained in the fermentation process of blue corn flour (*Zea mays* L.) with *Colletotrichum gloeosporioides* and of the flour without being subjected to any fermentation process.

Sample	Proportions (mL/g)	Humidity (g/100 g)	Ashes (g/100 g)	Proteins (g/100 g)	Lípids (g/100 g)	Carbohydrates (g/100 g)
Blue Corn Flour (*Zea mays* L.)	N. A.	9.19 ± 0.04	1.79 ± 0.00	8.67 ± 0.30	6.66 ± 0.03	73.67 ± 0.22
25 H	70-30	63.78 ± 0.28	1.01 ± 0.06	5.17 ± 0.68	5.58 ± 0.39	24.43 ± 0.01
25 F	80-20	84.75 ± 0.19	0.85 ± 0.03	5.30 ± 0.60	6.51 ± 0.45	2.56 ± 0.02

25F: 80 mL of Czapek Dox and 20 g of blue corn flour; 25H: 80 mL of Czapek Dox and 20 g of blue corn flour.

**Table 2:** Solubility test of the gels obtained in the fermentation process of blue corn flour with *Colletotrichum gloeosporioides*.

Samples	Hexane (C_6_H_14_)	Ethyl Acetate (C_4_H_8_O_4_)	Ethanol (C_2_H_5_OH)	Distilled Water
25F_1_	INS	INS	S	S
25F_2_	INS	INS	S	S
25F_3_	INS	INS	S	S
25F_1_	INS	INS	S	S
25F_2_	INS	INS	S	S
25F_3_	INS	INS	S	S

INS: insoluble; S: soluble. 25F_1_: first sample of the triplicate; 25F_2_: second sample of the triplicate; 25F_3_: third sample of the triplicate.

**Table 3:** Determination of median lethal dose (LD_50_) of the gels obtained in the fermentation process of blue corn flour (*Zea mays* L.) with *Colletotrichum gloeosporioides*.

Sample	Concentration (mg/Kg)	Mortality	DL_50_ (mg/Kg)
25F	100	1 ± 1.09	682
	75	2 ± 0.89	
	50	2 ± 1.87	
	25	1 ± 1.41	
	10	1 ± 0.5	
25H	100	2 ± 0.44	601
	75	3 ± 0.44	
	50	3 ± 0.54	
	25	2 ± 0.44	
	10	2 ± 0.54	

25F: 80 mL of Czapek Dox and 20 g of blue corn flour; 25H: 80 mL of Czapek Dox and 20 g of blue corn flour.

**Table 4:** Factorial design of the amount of gel produced in samples from the fermentation of blue corn (*Zea mays* L.) flour with *Colletotrichum gloeosporioides*.

Origin of Variations	Sum of Squares	Degrees of Freedom	Mean Squares	Fvalue	Probability	Critical Value for F
Proportions	89.19	2	44.59	0.50	0.61	3.55
Time	89.19	2	44.59	0.50	0.61	3.55
Interaction	444.19	4	1.24	1.24	0.32	2.92
Within-group	1600.17	18				

## Data Availability

The data presented in this study are openly available in article.
